# Authors’ reply

**DOI:** 10.4103/0019-5413.77144

**Published:** 2011

**Authors:** Raju Rijal, Bikram Prasad Shrestha, Girish Kumar Singh, Mahipal Singh, Pravin Nepal, Guru Prasad Khanal, P Rai

**Affiliations:** *Department of Orthopedics, BPKIHS, Dharan, Nepal*; 2 *Public Health and Community Medicine Lucknow, India*; 1 *CSSM, Lucknow, India*

Sir,

We thank you for the interest and raising these concerns ^1^on our paper.^2^

The existing recommendations favoring Ponseti method were based on plausibility. Time-related improvement quantifiable by standard easily reproducible outcome measure like Pirani scores was not documented, nor a comparable trial of both the methods was done. The data-based evidence collected from a rigorous randomized controlled trial (RCT) is a must to justify propogating Ponseti. For an RCT, the assumption that the two methods are equal despite the plausibility-based peer review that one is better justifies random allocation to both groups. True absence of an RCT in 2005 was a strong justification for the study. The project was approved by institutional ethics committee.

Sample size is a matter of concern in negative studies. Since the study was a part of research for a Master of Orthopedic Surgery degree, hence time bound, no commitment to a particular sample size was logistically possible. Moreover, there were no reliable estimates on numbers (effect sizes and variances in context of the study population) needed for sample size calculations.

Allocation concealment was not possible because some of the Ponseti feet carried the scar of tenotomy. However, an objective method of measurement like Pirani score is robust against measurement bias due to lack of blinding. Written informed consent was taken in every case.

Here 49 patients reported in the recruitment period for the study, 38 were eligible, 22 bilateral. Thus 60 feet were randomly allocated to the two groups. (In 12 bilateral patients, one foot went to Ponseti and one to Kite’s paired analysis). In 10 bilateral cases, both feet in 6 went to Kite’s method and 4 to Ponseti. In 16 unilateral cases, 6 went to Kite’s and 10 to Ponseti. There were no drop outs, loss to follow-up, or failures at 10 weeks in either group. “Follow-up was done at weekly interval for 10 weeks” mentioned in the fourth paragraph, page 203 of material and methods.

A paired biological situation, two feet of same patient allocated to two treatments, has to be analyzed as a paired situation because the results on one foot are more dependent (*F* > 3) on the results of the other foot (same genetics, age, rigidity, rate of growth, age and duration of intervention, etc. are the causes of dependence) as compared to a situation when the feet being compared belong to different patients. This would do nothing to alpha error if the results between paired and unpaired tests are not pooled.

*P* values have more information than confidence intervals. The randomized controlled superiority trial supporting Ponseti method over Kite’s method would have assumed that Ponseti method could not have been inferior, a biased conclusion not appropriate methodology wise given that the superiority of Ponseti method was only a popular opinion at the time of starting the study.

We again profusely thank you for the time invested in our study.
